# Improving Parental Health Literacy in Primary Caregivers of 0- to 3-Year-Old Children Through a WeChat Official Account: Cluster Randomized Controlled Trial

**DOI:** 10.2196/54623

**Published:** 2024-07-04

**Authors:** Yun Li, Qiuli Xiao, Min Chen, Chunhua Jiang, Shurong Kang, Ying Zhang, Jun Huang, Yulin Yang, Mu Li, Hong Jiang

**Affiliations:** 1 Department of Child Health Care, Shanghai Minhang Maternal and Child Health Care Hospital, Shanghai, China; 2 Department of Maternal, Child and Adolescent Health, School of Public Health, NHC Key Laboratory of Health Technology Assessment, Fudan University, Shanghai, China; 3 Shanghai Center for Women and Children’s Health, Shanghai, China; 4 School of Public Health, The University of Sydney, Sydney, Australia; 5 China Studies Centre, The University of Sydney, Sydney, Australia

**Keywords:** health literacy, WeChat, cluster randomized controlled trial, RCT, randomized, controlled trial, controlled trials, parental, parenting, parents, parent, China, Chinese, mHealth, mobile health, app, apps, applications, pediatric, pediatrics, paediatric, paediatrics, infant, infants, infancy, baby, babies, neonate, neonates, neonatal, newborn, newborns, toddler, toddlers

## Abstract

**Background:**

Parental health literacy is important to children’s health and development, especially in the first 3 years. However, few studies have explored effective intervention strategies to improve parental literacy.

**Objective:**

This study aimed to determine the effects of a WeChat official account (WOA)–based intervention on parental health literacy of primary caregivers of children aged 0-3 years.

**Methods:**

This cluster randomized controlled trial enrolled 1332 caregiver-child dyads from all 13 community health centers (CHCs) in Minhang District, Shanghai, China, between April 2020 and April 2021. Participants in intervention CHCs received purposefully designed videos via a WOA, which automatically recorded the times of watching for each participant, supplemented with reading materials from other trusted web-based sources. The contents of the videos were constructed in accordance with the comprehensive parental health literacy model of WHO (World Health Organization)/Europe (WHO/Europe). Participants in control CHCs received printed materials similar to the intervention group. All the participants were followed up for 9 months. Both groups could access routine child health services as usual during follow-up. The primary outcome was parental health literacy measured by a validated instrument, the Chinese Parental Health Literacy Questionnaire (CPHLQ) of children aged 0-3 years. Secondary outcomes included parenting behaviors and children’s health outcomes. We used the generalized linear mixed model (GLMM) for data analyses and performed different subgroup analyses. The β coefficient, risk ratio (RR), and their 95% CI were used to assess the intervention’s effect.

**Results:**

After the 9-month intervention, 69.4% (518/746) of caregivers had watched at least 1 video. Participants in the intervention group had higher CPHLQ total scores (β=2.51, 95% CI 0.12-4.91) and higher psychological scores (β=1.63, 95% CI 0.16-3.10) than those in the control group. The intervention group also reported a higher rate of exclusive breastfeeding (EBF) at 6 months (38.9% vs 23.44%; RR 1.90, 95% CI 1.07-3.38) and a higher awareness rate of vitamin D supplementation for infants younger than 6 months (76.7% vs 70.5%; RR 1.39, 95% CI 1.06-1.82). No significant effects were detected for the physical score on the CPHLQ, breastfeeding rate, routine checkup rate, and children’s health outcomes. Furthermore, despite slight subgroup differences in the intervention’s effects on the total CPHLQ score and EBF rate, no interaction effect was observed between these subgroup factors and intervention factors.

**Conclusions:**

Using a WHO literacy model–based health intervention through a WOA has the potential of improving parental health literacy and EBF rates at 6 months. However, innovative strategies and evidence-based content are required to engage more participants and achieve better intervention outcomes.

## Introduction

A child’s health and well-being are critical in the first 3 years of his or her life, over the short and long term [1,2]. During this period, a caregiver’s health literacy is crucial in the child’s development and health [3]. WHO (World Health Organization)/Europe (WHO/Europe) has proposed a 12D model that identifies 4 competencies for health literacy. These competencies include accessing, understanding, appraising, and applying health information across 3 domains: health care, disease prevention, and health promotion [4]. Studies have shown that caregivers with low health literacy were more likely to engage in risky parenting behaviors, such as not practicing breastfeeding (BF), not administering medications to children as prescribed, and allowing children long screen time [5-7]. These can lead to poor health outcomes for their children, such as poor disease management and high emergency service usage [8-10]. Despite this, few studies have evaluated the effectiveness of interventions aiming to improve parental health literacy.

The 2020 Chinese Health Literacy Surveillance Report revealed that only 35.9% of Chinese residents had basic health literacy [11]. Health professionals in China were confronted with a limited health workforce and high demand for childcare services [12]. In-person communication has also been restricted due to the COVID-19 pandemic, making it challenging for caregivers to access reliable health information [13]. Therefore, there is an urgent need for feasible and effective alternative approaches to deliver health promotion and support for caregivers of young children.

Social media has become a popular platform for individuals to obtain and share health-related information in recent years [14-18]. WeChat, one of China’s largest social media platforms, had over 1.3 billion monthly active users worldwide by the end of 2023 [19]. WeChat offers several innovative function modules, including WeChat official accounts (WOAs), which enable users to access information, services, and subscriber interaction [20]. A recent national survey showed that one-third of respondents regularly accessed health information via WeChat and nearly two-thirds considered WOAs a feasible medium for accessing health education material [21]. Studies have shown that using WeChat or WOAs for health intervention could significantly improve satisfaction, accessibility, and convenience compared to traditional health education [22,23]. By integrating diverse forms of information, including text, images, videos, and links to digital books and web pages, WeChat and WOAs have the potential to enhance parental health literacy. WeChat’s health education campaign for pregnant women has been proven effective in promoting exclusive breastfeeding (EBF) [24]. A WeChat-based family health education program also significantly raised parents’ awareness of children’s illness prevention [25]. However, most existing studies have only addressed limited aspects of health literacy. Our WOA intervention is grounded in the WHO/Europe comprehensive health literacy model. It aims to enhance parental health literacy, and its effectiveness is evaluated through a cluster randomized controlled trial among caregivers of children aged 0-3 years in an urban setting in China.

## Methods

### Study Design

According to a prespecified study protocol, a cluster randomized controlled trial was conducted from April 7, 2020, to April 20, 2021, in Minhang District, Shanghai, China [26]. All 13 Minhang District community health centers (CHCs) were enrolled and randomly allocated to the intervention or control group through random sequence generation, with 1332 eligible caregiver-child dyads participating in the trial. The CHCs were responsible for providing child health care services in their catchments for children from birth to 3 years of age.

### Participants

Since we planned a 9-month intervention, we recruited primary caregivers of children aged 0-3 years. The primary caregiver herein refers to the person who provided primary care to the child, including the child’s mother, father, grandmother, grandfather, and other caregivers including nannies. One caregiver at most was recruited within each family. In case of families with multiple children, the participating caregiver would designate only one of them to be involved in this study and provide their unique health record number.

When caregiver-child dyads attended child health care in CHCs, the CHCs’ staff approached and informed them about the study. Caregivers were eligible if they were adults (older than 18 years) and primary caregivers of children aged 0-3 years, completed at least the third grade of primary school, owned a smartphone and an individual WeChat account, were able to communicate verbally or in writing, and planned to stay in the recruitment area for at least a year. Immediately after recruitment, all participating caregivers were invited to subscribe to the designed WOA for data collection in both groups and intervention delivery only for the intervention group.

### Blinding

Due to the nature of the intervention, service providers and participants were not blinded to the group allocation. Immediately after baseline data collection, group allocation was revealed to the participants.

### Interventions

#### Contents of the WOA-Based Intervention

Contents of the intervention videos were developed in accordance with 12 subdimensions of health literacy by WHO/Europe [4]. Through literature review and expert consultation, 15 key topics about children’s health and development (10 physical and 5 psychological) were generated and categorized into 3 domains, including health care, disease prevention, and health promotion. Each key topic was designed to improve caregivers’ 4 types of capacity in accessing, understanding, appraising, and applying child health information. The detailed logic model for this WOA-based intervention program has been prespecified in our protocol [26].

#### The WOA Platform

**Figure 1.**
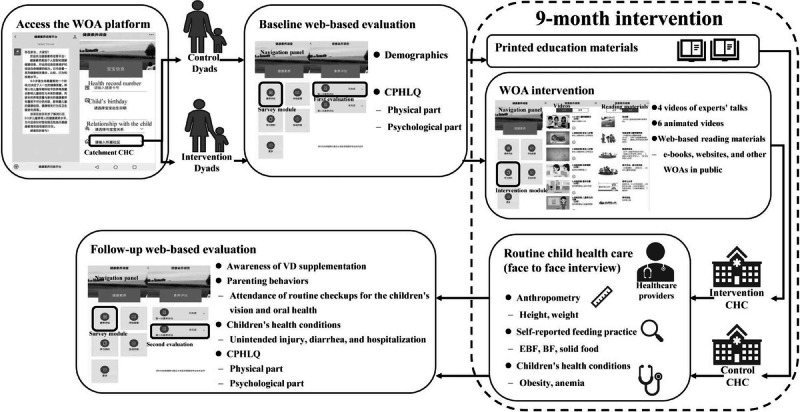
Participants’ journey through the scientific parenting platform of the WeChat official account (WOA)–based intervention. BF: breastfeeding; CHC: community health center; CPHLQ: Chinese Parental Health Literacy Questionnaire; EBF: exclusive breastfeeding; VD: vitamin D.

The Scientific Parenting platform, specifically designed for this WOA-based intervention, was integrated into the WeChat app. Participants were invited to subscribe and register upon providing informed consent. The registration involved entering their child’s unique health record number, date of birth, relationship with the child, and catchment CHCs. Once participants logged in, the platform automatically assigned them to different functions based on their catchment CHCs. All participants had access to the web-based survey module, while only those in the intervention CHCs could see and access the intervention module. Two electronic questionnaires were automatically delivered to each participant via the web-based survey module, one at recruitment and the other 9 months later. Figure 1 shows a detailed illustration of the participants’ journey through the Scientific Parenting platform.

#### Intervention Group

The intervention package was provided in the intervention module, consisting of videos developed on the basis of the previously mentioned WHO/Europe health literacy model. The videos included 4 video clips of experts’ talks between 13 and 33 minutes and 6 animated video clips of 1 to 5 minutes, totaling 2 hours. During the 9-month intervention, participants could self-navigate, select topics that they were interested in, and decide the order and pace of material they read or watch. Participants could watch the videos repeatedly, and the number of times the videos were watched was recorded automatically by the platform in each user’s account. Additionally, we provided participants with reading material from other trusted web-based sources as supplemented material to videos. Participants could access this web-based reading material by clicking on links, such as e-books, websites, and other WOAs in public. Furthermore, the contents were closely related to key topics of the videos, and the web-based reading material also covered other topics such as early education, healthy home environment, etc.

#### Control Group

Participants in the control group received printed educational material as an extension to routine child health care when they took their children for health checkups. The contents of the printed material resembled those of the videos delivered to the intervention group. The printed educational material is also detailed in our protocol [26].

Both groups received routine child health care services, including weight and length measurements, self-reported child feeding practice and dietary intake, and checkups of children’s health conditions [27].

### Outcome Measures

The primary outcome measured was the change in total scores of parental health literacy (physical and psychological) using a validated questionnaire [28,29]. Secondary outcomes included (1) parenting behaviors, such as the EBF rate at 6 months, any BF rate at 12 months, the consumption rate of iron-fortified staple foods at 6 months, and the rate of routine checkup of the child’s vision and oral health; (2) the proportion of participants’ awareness of vitamin D (VD) supplementation for infants aged 0 to 6 months; (3) children’s anthropometric measures, including weight-for-length *z* score, length-for-age *z* score, weight-for-age *z* score, BMI-for-age *z* score, and head circumference-for-age *z* scores; and (4) children’s health outcomes, such as the incidence of anemia, unintended injuries, diarrhea, obesity, and hospitalization.

### Data Collection

All participants completed the baseline survey through the web-based survey module on the WOA platform. The final survey will automatically be made available to the participants 9 months after the baseline survey.

In the baseline survey, we collected demographics and health-related variables, including participants’ relationship with their children, education level, source of parental information, family income, number of children in the family, children’s age, gender, and Hukou (the Chinese official permanent residency registration by location, which is closely linked with social welfare and administration [26,27]).

Parental health literacy was measured using a validated questionnaire, the Chinese Parental Health Literacy Questionnaire (CPHLQ) of children aged 0-3 years, at baseline and the end line [28,29]. The CPHLQ was designed by the research group based on the health literacy model proposed by WHO/Europe [4]. This questionnaire was validated among primary caregivers of children aged 0-3 years across different regions in China [28,30]. It consists of a 39-item subscale for physical health literacy and a 35-item subscale for psychological health literacy. Both the physical and psychological subscales were scaled on a score of 0-100, of which the total maximum score was 200.

In the final survey, we collected data on awareness of VD supplementation, parenting behaviors, including attendance in routine checkups for the children’s vision and oral health, and children’s health outcomes, such as unintended injury, diarrhea, and hospitalization. For awareness of VD supplementation, we asked the participants if they thought that administering VD supplements to their children after birth was necessary. For attendance in routine checkups, we asked if they had taken their children for regular visual and oral health checkups during the intervention. For children’s health outcomes, we asked about the occurrence of unintended injury, diarrhea, and hospitalization in the past 9 months. Participants’ engagement in the intervention, the frequency of video watching by participants, was automatically tracked by a function embedded in the WOA platform. However, due to administration rights, we could not track participants’ accessing links to reading materials on other web-based platforms.

Children living in the project district were required to take routine health checkups at specific ages: 1, 2, 4, 6, 9, 12, 18, 24, 30, and 36 months. During these checkups, child health care providers at CHCs would conduct face-to-face interviews to collect data on various parameters such as EBF, BF, introduction of solid foods, anthropometric measures, and health conditions such as obesity and anemia [31]. Project researchers would later extract all relevant health checkups’ data from baseline to 1 month after the 9-month intervention. Children younger than 6 months who were exclusively breastfed at baseline were eligible for the EBF analysis. Similarly, children younger than 6 months who had not been introduced to solid foods at baseline were eligible for the analysis of supplementation of iron-fortified staple foods at 6 months. Children younger than 12 months who were still breastfed at baseline were eligible for the BF analysis. For the analysis of anthropometric measures and health conditions such as obesity and anemia, health checkup data closest to the final web-based evaluation survey were used, and baseline values were included as covariates.

### Sample Size Calculation

We assumed an intervention effect size of 15 points for total scores on the CPHLQ, a coefficient of variation of 0.2, and an intracluster correlation coefficient of 0.05 [28]. A total of 1183 caregivers from 13 CHCs (clusters), with a mean cluster size of 91 per CHC, was estimated to detect the assumed effect size with 90% power at a 5% significance level. We also considered an approximately 20% rate of loss to follow-up.

### Statistical Analysis

We conducted descriptive statistical analyses for all outcomes, presenting continuous variables as mean (SD) or median (IQR) values and categorical variables as proportions (%). To compare differences between the 2 groups, we used independent *t* tests for continuous variables and chi-square tests for categorical variables. Anthropometric indicators were calculated using the WHO Anthro (version 3.2.2) [32]. To compare within-group differences (mean change from baseline) between the baseline and at the end of 9 months of the intervention, we used the paired *t* test.

To assess the intervention’s effects, we performed intention-to-treat analyses and generalized linear mixed models (GLMMs), as recommended for cluster randomized trials [33]. We used multiple imputations to handle missing values at the end of the trial. The GLMMs included CHC-level random intercepts to account for the correlation due to the clustering of participants within CHCs. For each outcome analysis, the model was adjusted for demographic characteristics. Additionally, the baseline CPHLQ score, anthropometric *z* score, and status of obesity and anemia were included in the model for evaluation of the intervention effects. By controlling the correlation between baseline and follow-up values, we avoided the need for time or a time×group interaction term to interpret intervention effects [34]. Given the relatively small number of clusters, Satterthwaite correction was used in GLMM to maintain an appropriate type I error [35]. Satterthwaite correction allowed us to estimate the degree of freedom on the basis of the residual variances at different variable levels [36].

We reported the intervention effect using β (95% CI) and *P* values for continuous outcomes. We used risk ratios (RRs), their 95% CIs, and *P* values to describe the intervention effect for categorical outcomes.

To identify any potential modifications, we conducted subgroup and interaction analyses for the total score on the CPHLQ and the EBF rate at 6 months. Participants were stratified into diverse subgroups based on their relationship with the child (mothers vs others), the child’s Hukou (Shanghai vs others), whether they had 1 child or more, the participant’s education level (university or higher vs less than university education), and source of parental information access (social media vs others). For the total score on the CPHLQ, we presented the β (95% CI) of GLMMs within each subgroup and the *P* value for interaction items of GLMMs in the overall group. For the EBF rate at 6 months, we presented the RR (95% CI) of GLMMs within each subgroup. Additionally, we used the multiplicative scale for multiplicative interaction and the relative excess risk due to interaction, attributable proportion due to interaction, and synergy index for additive interaction in the overall GLMMs.

Results with a type I error rate of *P*<.05 in 2-sided tests were considered statistically significant. Statistical analysis was performed using R statistical software (version 4.1.3; The R Foundation).

### Ethical Considerations

The trial was approved by the ethics committee of Shanghai Minhang District Maternal and Child Health Hospital (approval number #2020-KS-01) and registered with the Chinese Clinical Trial Registry (#ChiCTR2000031711). All trial participants provided their written informed consent during the recruitment process.

## Results

### Study Participation

**Figure 2.**
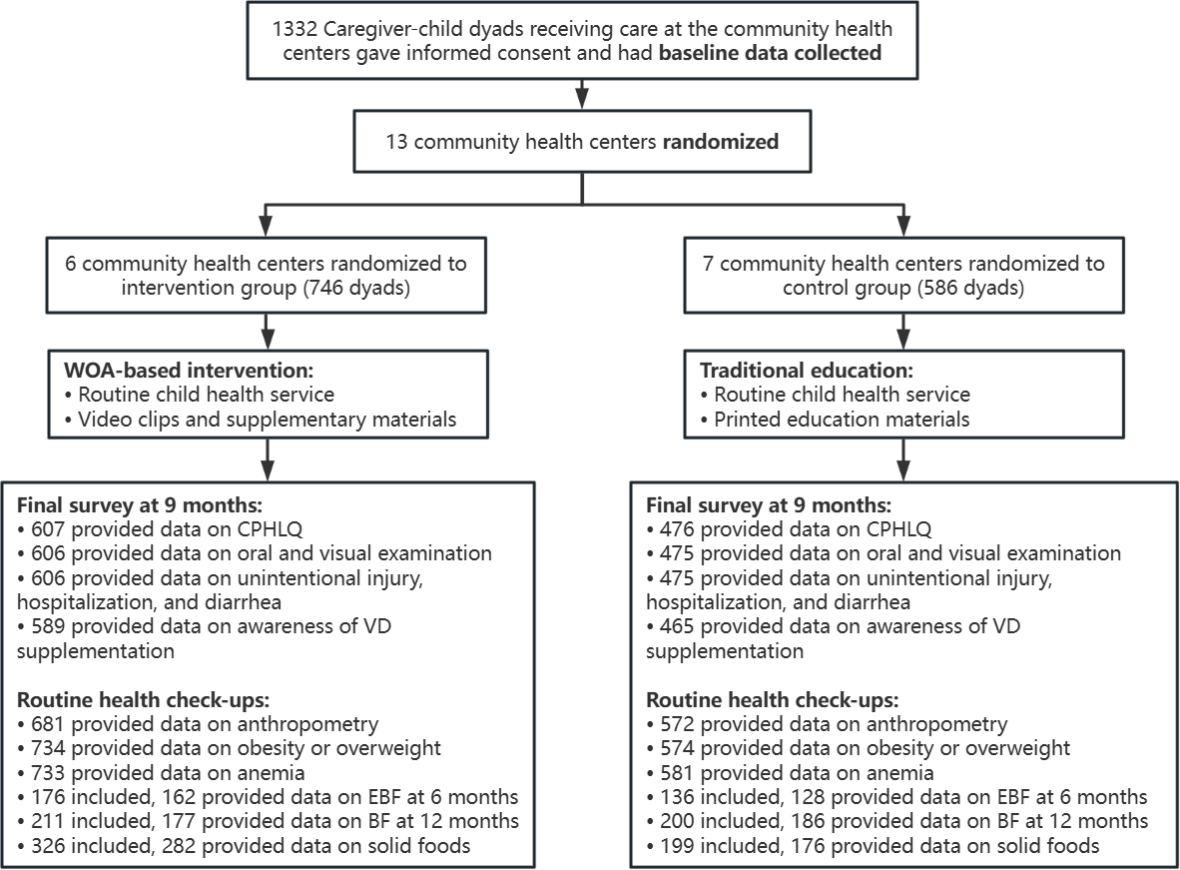
Participant recruitment and retention of the WeChat official account (WOA)–based intervention. BF: breastfeeding; CPHLQ: Chinese Parental Health Literacy Questionnaire; EBF: exclusive breastfeeding; VD: vitamin D.

A total of 1332 caregiver-child dyads from 13 CHCs participated in the study. Six CHCs containing 746 dyads were randomly assigned to the intervention group, and 7 CHCs containing 586 dyads to the control group (Figure 2).

According to the baseline information, among 1332 participants, 81.7% (1088/1332) of primary participants were mothers and 82.9% (1104/1332) of participants had college or above education. The majority (92.7%, 1235/1332) of participants accessed parenting information from other social media sites (Table 1). Among all children aged 0-3 years, 71.2% (948/1332) of them were the only children in the family, 48.4% (645/1332) of them were boys, and 62.0% (826/1332) of them were in the Shanghai Hukou (Table 1).

**Table 1.** Baseline characteristics of the participants in the WeChat official account (WOA)–based intervention (April 7, 2020, to April 20, 2021, in Shanghai).

Variable		Control (n=586)	Intervention (n=746)	Total (N=1332)	*P* value^a^
Child’s age (months), mean (SD)		8.97 (6.77)	8.56 (7.17)	—^b^	.28
**Child’s gender, n (%)**					.83
	Male	284 (48.5)	361 (48.4)	645 (48.4)	
	Female	302 (51.5)	385 (51.6)	687 (51.6)	
**Relationship with the child, n (%)**					.051
	Mother	465 (79.4)	623 (83.5)	1088 (81.7)	
	Father or others	121 (20.6)	123 (16.5)	244 (18.3)	
**Child’s Hukou, n (%)**					*<.001^c^*
	Shanghai	414 (70.6)	412 (55.2)	826 (62.0)	
	Other provinces	172 (29.4)	334 (44.8)	506 (38.0)	
**One-child or not, n (%)**					.07
	Yes	432 (73.7)	516 (69.2)	948 (71.2)	
	No	154 (26.3)	230 (30.8)	384 (28.8)	
**Participants’ education, n (%)**					*<.001*
	Junior school and below	20 (3.4)	62 (8.3)	82 (6.2)	
	High school	57 (9.7)	89 (11.9)	146 (11.0)	
	University or higher	509 (86.9)	595 (79.8)	1104 (82.9)	
**Family monthly income per capita (in RMB** ^**d**^ **), n (%)**					.75
	<4500	49 (8.4)	73 (9.8)	122 (9.2)	
	4500-7500	126 (21.5)	161 (21.6)	287 (21.5)	
	7500-12,500	186 (31.7)	191 (25.6)	377 (28.3)	
	≥12,500	171 (29.2)	243 (32.6)	414 (31.2)	
	I do not know	54 (9.2)	78 (10.5)	132 (9.9)	
**Source of parental information, n (%)**					*.04*
	Social media	553 (94.4)	682 (91.4)	1235 (92.7)	
	Others	33 (5.6)	64 (8.6)	97 (7.3)	
**Feeding mode, n (%)**					.47
	Exclusive breastfeeding	152 (26.0)	185 (24.9)	337 (25.4)	
	Mixed feeding	183 (31.3)	256 (34.5)	441 (33.2)	
	Artificial feeding	248 (42.7)	302 (40.6)	550 (41.4)	

a *P* values for comparing the intervention and control groups.

b Not applicable.

c Italicized values are significant at *P*<.05.

d 1 RMB=US $0.14.

There was no significant difference between the intervention and the control group regarding participants’ relationship with their child, the child’s age and gender, the number of children in the family, family monthly income, and child feeding mode (*P*>.05). However, compared to the control group, fewer participants in the intervention group had a university or higher level of education, children with Shanghai Hukou, and received information from other social media sites (*P*<.05).

### Participants’ Adherence to the Intervention Program

During the 9-month intervention program, the WOA platform automatically tracked the frequency of participants watching the videos. Of 746 participants in the intervention group, 69.4% (518/746) had watched at least 1 video. Participants who watched the videos tended to have younger children (8.12 vs 9.54 months, *P*=.01), not have a Shanghai Hukou (49.8% vs 33.3%, *P*<.001), be the children’s mothers (85.9% vs 78.1%, *P*=.01), have an education below university level (23.0% vs 14.0%, *P*=.007), and remain in the study (83.4% vs 76.8%, *P*=.04), compared to those who never watched any video during the intervention (Table S1 in Multimedia Appendix 1).

At each video level, less than 50% (range 14.9%-46.5%) of participants in the intervention group had ever watched the video. The 3 videos with the highest watching rate were scientific feeding guidance (46.5%), childhood pneumonia identification (41.7%), and childhood obesity prevention (41.6%). The average number of times viewing each video clip ranged from 0.14 to 1.35. The most frequently viewed video clips were scientific feeding guidance (1.35 times), unintentional injury prevention (0.91 times), and essential information on health care for children aged 0 to 3 years (0.81 times).

### Retention of the Study Participants

At the end of 9 months, the retention rates of the intervention and the control groups did not differ significantly (81.4% vs 81.2%, *P*=.95). Compared to participants enrolled at baseline, those lost to follow-up tended to be the child’s father or other caregivers (18.3% vs 30.1%, *P*<.001). Compared to participants who remained in the study, they also tended to be child’s father or other caregivers (15.6% vs 30.1%, *P*<.001) and have a child without a Shanghai Hukou (36.7% vs 43.8%, *P*=.04; Table S2 in Multimedia Appendix 1). Please refer to Multimedia Appendix 2 for a detailed missing data report of this study.

### Intervention Effect

#### Parental Health Literacy

**Figure 3.**
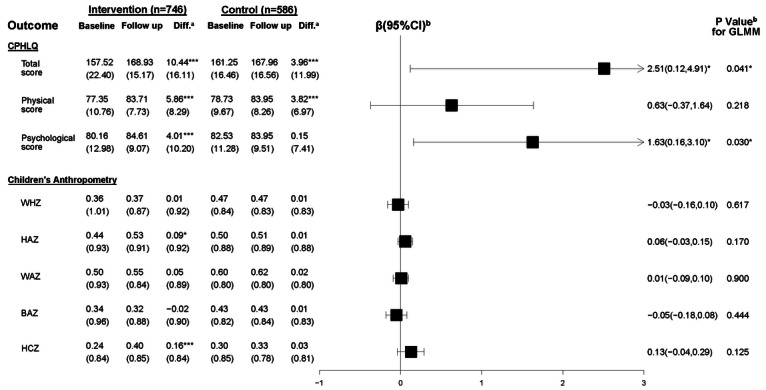
Effect of the WeChat official account (WOA)–based intervention (April 7, 2020, to April 20, 2021, in Shanghai) on the Chinese Parental Health Literacy Questionnaire (CPHLQ) and children’s anthropometric measures. ^a^*P* values describing the changes from baseline to follow-up in the intervention or control group. ^b^The results of the generalized linear mixed model (GLMM) with the group as a fixed factor, community health centers (CHCs) as the random intercept, follow-up values as outcomes, and baseline values, caregiver’s relationship with the child, education level, the child’s gender, Hukou, whether they are the only child or not, and source of parental information access as covariates. BAZ: BMI-for-age *z* score; HAZ: length-for-age *z* score; HCZ: head circumference–for-age *z* score; WAZ: weight-for-age *z* score; WHZ: weight-for-length *z* score. *.01<*P*≤.05; **.001<*P*≤.01; ***P≤.001.

The results of parental health literacy are presented in Figure 3. At the final evaluation, both the intervention and control groups showed increased scores on the CPHLQ compared to baseline. The intervention group had increased total, physical, and psychological part scores on the CPHLQ by 10.44, 5.86, and 4.01 points, the control group had an increase by 3.96, 3.82, and 0.15 points, respectively, compared to baseline. Using the GLMM and controlling for the baseline score and other potential confounders, we observed a significant intervention effect on the total CPHLQ scores (β=2.51, 95% CI 0.12-4.91) and psychological scores (β=1.63, 95% CI 0.16-3.10), while there was no significant difference in the physical parental health literacy score.

**Figure 4.**
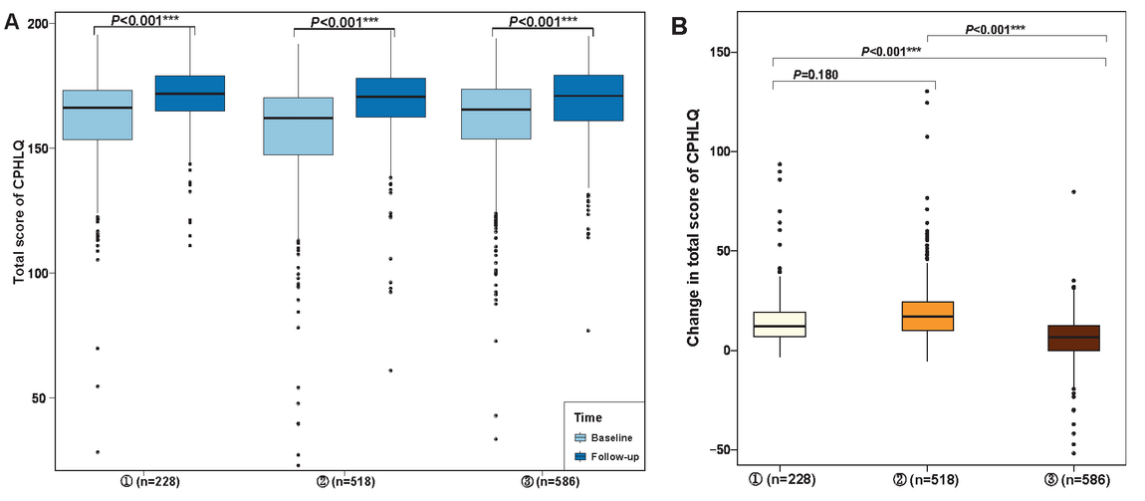
Comparisons of total score on the Chinese Parental Health Literacy Questionnaire (CPHLQ) among participants with different adherent statuses in the WeChat official account (WOA)–based intervention (April 7, 2020, to April 20, 2021, in Shanghai). ^a^*P* values describing differences in the total score from baseline to follow-up using the paired *t* test. ^b^*P* values describing the difference in change in the total score between participants with different adherent statuses using the *t* test. Participants with different adherent statuses: ① those in the intervention group who had never watched videos; ② those in the intervention group who had watched at least 1 video; and ③ those in the control group. ***P≤.001.

We analyzed the effect of watching at least 1 intervention video on CPHLQ scores (Figures4  5). Within the intervention group, both participants who had not watched videos and those who watched at least 1 video demonstrated an increased total score. Using the GLMM, compared to participants who watched at least 1 video, those in the control group had a significantly lower total score (β=2.68, 95% CI 0.20-5.17) and psychological score (β=1.72, 95% CI 0.13-3.32), after controlling for the baseline score and other potential confounders.

**Figure 5.**
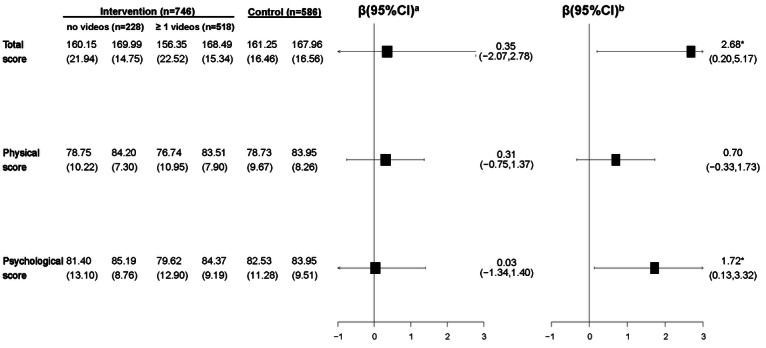
Effect of watching at least one video of the WeChat official account (WOA)–based intervention (April 7, 2020, to April 20, 2021, in Shanghai) on participants’ total scores on the Chinese Parental Health Literacy Questionnaire (CPHLQ). ^a^The results of the generalized linear mixed model (GLMM) analyzing the difference between caregivers who have not watched the videos and those who watched at least 1 video within the intervention group. ^b^The results of the GLMM analyzing the difference between caregivers in the control group and caregivers who have not watched videos in the intervention group. *.01<*P*≤.05.

**Figure 6.**
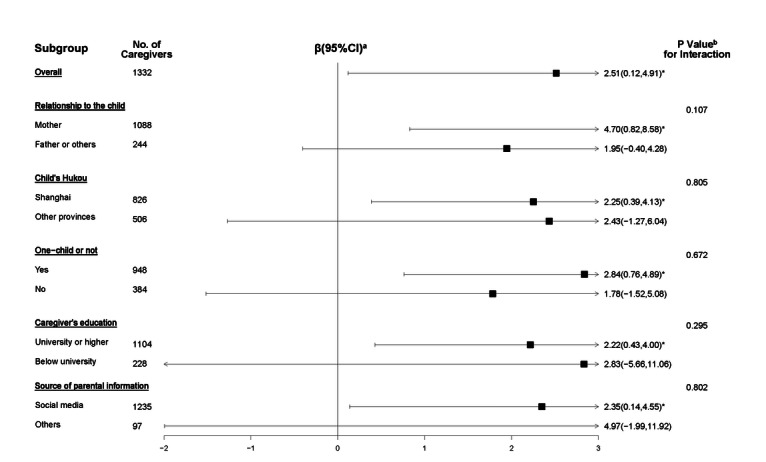
Subgroup analyses of the effect of the WeChat official account (WOA)–based intervention (April 7, 2020, to April 20, 2021, in Shanghai) on participants’ total score on the Chinese Parental Health Literacy Questionnaire (CPHLQ). ^a^The results of the generalized linear mixed model (GLMM) within each subgroup with the group as a fixed factor, community health centers (CHCs) as the random intercept, follow-up values as outcomes, and baseline values, caregiver’s relationship with their child, education level, child’s gender, Hukou, being a single child or not, and source of parental information as covariates. ^b^*P* value for the interaction term of the GLMM within the overall group. *.01<*P*≤.05.

Despite slight differences in the intervention’s effects on the total score on the CPHLQ among different subgroups, no interaction effect was observed between these subgroup factors and the intervention (Figure 6).

#### Parenting Behaviors

**Figure 7.**
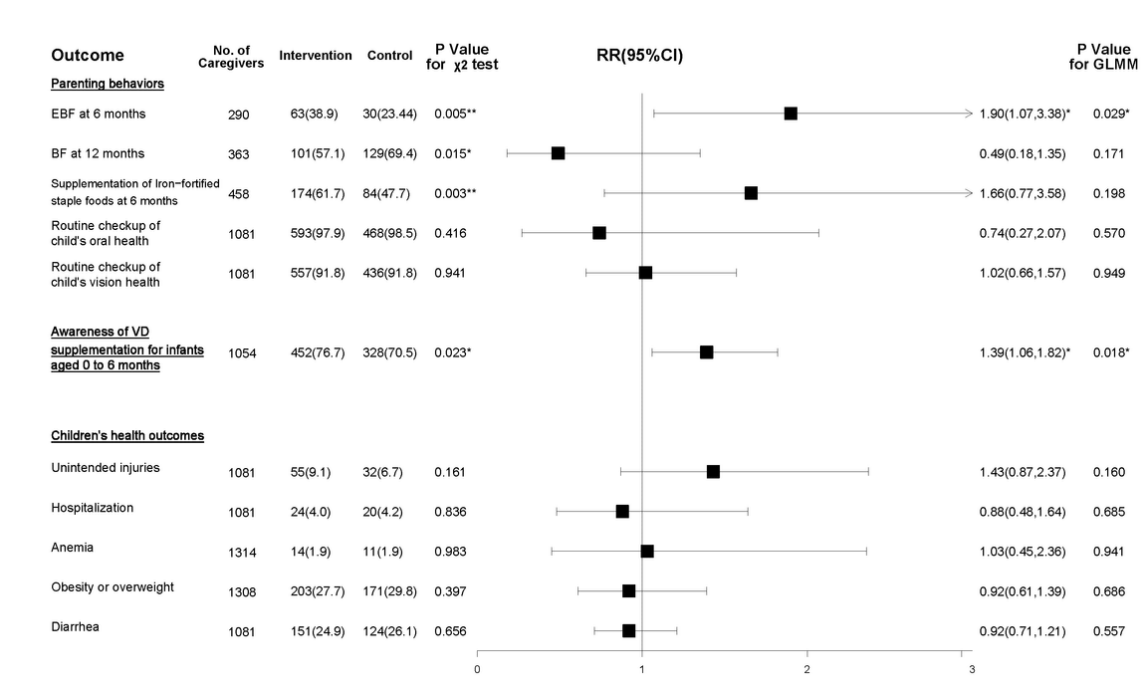
Effect of the WeChat official account (WOA)–based intervention (April 7, 2020, to April 20, 2021, in Shanghai) on parenting behaviors, awareness, and children’s health outcomes. The risk ratio (RR) and 95% CI values describe the results of the generalized linear mixed model (GLMM) with the group as a fixed factor, community health centers (CHCs) as the random intercept, follow-up values as outcomes, and baseline values for caregiver’s relationship with their children, education level, child’s gender, Hukou, whether they are an only child or not, and source of parental information access as covariates. BF: breastfeeding; EBF: exclusive breastfeeding; VD: vitamin D. *.01<*P*≤.05; **.001<*P*≤.01; ***P≤.001.

The effect of the intervention on parenting behaviors is illustrated in Figure 7. According to the GLMMs controlled for potential confounders, the intervention group demonstrated a significantly higher EBF rate at 6 months than the control group (38.9% vs 23.44%; RR 1.90, 95% CI 1.07-3.38). Additionally, there were no significant between-group differences in the BF rate at 12 months, iron-fortified staple food supplementation at 6 months, and routine checkup of the child’s vision and oral health.

**Figure 8.**
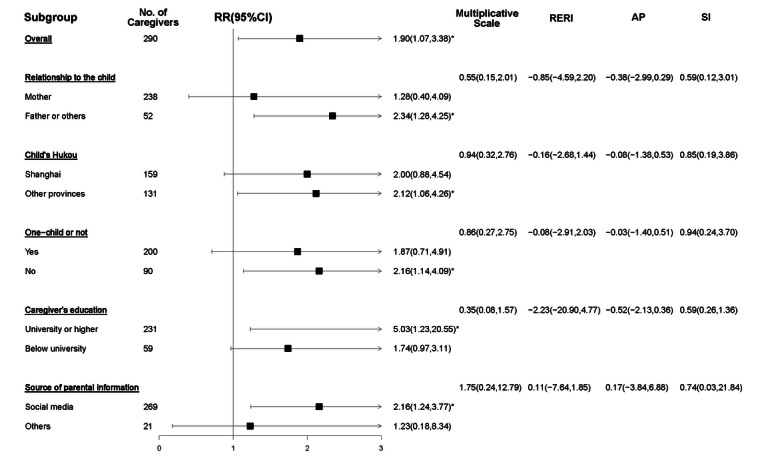
Subgroup analyses of the effects of the WeChat official account (WOA)–based intervention on participants’ exclusive breastfeeding (EBF) rate at 6 months. The risk ratio (RR) and 95% CI values describe the results of the generalized linear mixed model (GLMM) within each subgroup with the group as a fixed factor, community health centers (CHCs) as the random intercept, follow-up values as outcomes, and baseline values of caregiver’s relationship with the children, education level, child’s gender, Hukou, whether they are an only child or not, and source of parental information as covariates. The multiplicative scale described the multiplicative interaction between subgroup factors and intervention, while the relative excess risk due to the interaction (RERI), attributable proportion due to the interaction (AP), and synergy index (SI) describe the additive interaction. *.01<*P*≤.05.

The results of subgroup analyses on EBF at 6 months are presented in Figure 8. Neither multiplicative nor additive interaction effects were observed between the subgroup factors and the intervention.

#### Awareness of VD Supplementation After Birth

The results of VD supplementation awareness are presented in Figure 7. After controlling for potential confounders, the GLMM showed that the intervention group had a significantly higher awareness rate of VD supplementation after birth than the control group (76.7% vs 70.5%; RR 1.39, 95% CI 1.06-1.82).

#### Children’s Anthropometric and Health Outcomes

The results of children’s anthropometric and health outcomes are respectively presented in Figures 3 and 7. Using the adjusted GLMM, no significant intervention effects were observed in children’s anthropometric and health outcomes between the intervention and control groups.

## Discussion

### Principal Findings

We developed a digital parenting health literacy intervention of purposefully designed videos, supplemented with reading material from other web-based platforms and evaluated the effectiveness of the intervention. The rate of viewing at least 1 intervention video was nearly 70%. The 9-month WOA-based intervention significantly improved the health literacy score among caregivers of 0‐ to 3-year-old children, EBF rate at 6 months, and their awareness of VD supplementation for young children.

### Comparison With Previous Studies

Digital technology has been widely used as an educational tool for health information delivery [37,38]. WeChat is a popular Chinese instant messaging and social media application. It has also been used to overcome temporal and geographical constraints to deliver health-related information and provide health support [39]. Evidence has shown the effects of WeChat-based interventions on improving specific parenting knowledge or practices. For example, perioperative health education via a WeChat platform effectively enhanced parents’ knowledge of care for children with heart diseases [23]. A WeChat-based parenting training proved promising in improving parenting attitudes among mothers of children with autism during the COVID-19 pandemic [13]. A WeChat health education program in Qinghai, China, significantly increased the early-life EBF rate [24]. Our study results provide further evidence regarding the feasibility of improving parental health literacy via WeChat in a community setting.

The intervention group had significantly increased scores on the CPHLQ’s psychological component after the intervention and a significant group effect after controlling for baseline scores and other confounding factors. This finding suggests that the WOA-based intervention was superior in improving psychological health literacy compared to traditional paper-based health promotion interventions. A recent study supported our finding and reported that a WeChat-based program improved the psychospiritual well-being of patients with digestive cancers [40]. Psychological interventions delivered through WeChat were more effective probably because the contents were more diversified and engaging, such as text, images, audio, videos, and interactional games [20].

Since the caregivers’ educational level could be a significant determinant of parental health literacy [41], we performed subgroup analyses to evaluate the intervention’s effects among caregivers with different educational levels. Although we did not observe significant interaction effects, the results of subgroup analyses show that this WOA-based intervention might be effective only for caregivers with university-level or higher education. This could be possibly explained by the small proportion of caregivers with relatively lower education, only 13% of them with less than university-level education [42], which contributed to an insufficient sample size in the low-education subgroup analysis. Another reason might be that people with a higher education level were also equipped with higher eHealth literacy and thus had a better understanding of web-based information than others [43]. Therefore, future health literacy intervention research should focus on low education to further assess the effect of WOA-based interventions.

The intervention improved the EBF rate at 6 months. In China, many parents and caregivers believe that the right time for stopping EBF and introducing solid foods is approximately 4 months [31]. Although child health staff in Minhang District had promoted EBF for 6 months, at baseline, many caregivers were still unaware of the significance of the recommendation. In this study, we emphasized the importance of EBF for 6 months via a WOA-based intervention program, demonstrating an increase in the EBF rate in the first 6 months. No intervention effect was observed for any breastfeeding at 12 or 24 months. This could be explained by the influence of confounding factors, such as mothers returning to work before their children turn 6 months old and the lack of a supportive environment in the workplace [44]. Although the law mandates an hour of lactation leave per day for nursing mothers in the workplace, supports from society and the workplace for breastfeeding remain to be improved [45], especially in workplaces with a high female employment rate.

VD plays a crucial role in bone health and immunity and has been gaining growing attention for its role in children’s oral health [46,47]. During infancy, VD deficiency is associated with various disorders, such as dental caries and rickets [48]. In an animated video, we recommended that a liquid VD supplement of 400 international units per day begins within the first few days of life and continues throughout childhood, as suggested by the American Academy of Pediatrics [49] and the Chinese Dietary Guidelines for Children [50]. After the 9-month intervention, participants in the intervention group had a higher level of awareness of VD supplementation for infants aged 0-6 months than those in the control group. This finding is similar to that of a previous web-based intervention study [51].

Early screening for visual and oral disorders is an important content of routine health checkups for young children, which can identify related disorders, such as acquired blindness and dental caries [52,53]. Current evidence suggests that maternal education increased children’s visit to oral health care services [54]. Considering that social media has been widely used in health education [55], we hypothesized that a WOA-based intervention for caregivers could significantly improve children’s visit to routine checkups of visual and oral health. However, we did not find such an effect in our study. This might be due to the relatively low weight of the intervention contents on these topics compared to others such as nutrition. In addition, health education resources specified for children’s visual and oral health development need to be further strengthened in China to promote attendance in routine checkups [56].

### Limitations

Although this study has shown promising results in enhancing parental health literacy, some limitations should be acknowledged. First, it was conducted only in 1 district of Shanghai, which limits its generalizability to nonurban areas or populations with diverse demographics. Second, despite using random sequence generation for cluster randomization, there was an imbalance between groups in participant’s education and child’s Hukou, etc. This might be mainly attributed to the small number of clusters [57]. To minimize bias, we included potential confounders in the multivariate analysis and corrected *P* values by the Satterthwaite method. Future study involving more clusters are recommended to evaluate the intervention’s effect more accurately. Third, the low viewing rate of the intervention videos suggests a lack of learning motivation among participants. Given that the intervention was delivered in a real-world setting, this could reflect the true compliance of the health education intervention among caregivers in real life. Unfortunately, we could not collect data on participants’ exposure to supplementary web-based reading material due to the unavailability of administration rights to access the information. Lastly, child psychological development was not included as an outcome measure because psychological assessment is not routinely conducted in child health checkups because there is a need for specialized training, additional resources, and participant cooperation.

### Conclusions

We developed a comprehensive digital parental health literacy intervention program using WAOs. The intervention offered caregivers a reliable and trusted official source of parental health information. We demonstrated that the social media–based interventions delivered through a WOA could improve parental health literacy and parenting behaviors. Compared with traditional paper-based health materials, a WOA-based intervention can potentially be used for health education to address other public health challenges. Evidence-based content and innovative strategies are needed to engage more participants and achieve better intervention outcomes.

## Supplementary material

10.2196/54623 Multimedia Appendix 1 Participants’ adherence and retention to the intervention.

10.2196/54623 Multimedia Appendix 2 Missing data report for the WeChat official account–based intervention.

10.2196/54623 Checklist 1 CONSORT-eHEALTH (Consolidated Standards of Reporting Trials of Electronic and Mobile HEalth Applications and onLine TeleHealth) checklist (version 1.6.1).

## Abbreviations

BF: breastfeeding
CHC: community health center
CPHLQ: Chinese Parental Health Literacy Questionnaire
EBF: exclusive breastfeeding
GLMM: generalized linear mixed model
RR: risk ratio
VD: vitamin D
WHO: World Health Organization
WHO/Europe: World Health Organization–Europe
WOA: WeChat official account
