# Supplementary material for: Improving Parental Health Literacy in Primary Caregivers of 0- to 3-Year-Old Children Through a WeChat Official Account: Cluster Randomized Controlled Trial
Source: JMIR Public Health Surveill. 2024 Jul 4;10:e54623. doi: 10.2196/54623 (PMC11238142; doi:10.2196/54623)
Supplement: Multimedia Appendix 1 [file publichealth-v10-e54623-s001.docx]

## Multimedia appendix 1. Participants' adherence and retention to the intervention

Table S1. Baseline characteristics of the participants in the WOA-based intervention group with different adherent status (April 7, 2020, to April 20, 2021 in Shanghai)

| Variable | Intervention | No. of videos watched | | *P^a^* |
| --- | --- | --- | --- | --- |
|  |  | 0 videos | >=1 videos |  |
| No. of caregivers | 746 | 228 | 518 |  |
| Follow-up, n (%) | | | | 0.041 |
| No | 139 (18.6) | 53 (23.2) | 86 (16.6) |  |
| Yes | 607 (81.4) | 175 (76.8) | 432 (83.4) |  |
| Child's age (months), M (SD) | 8.56 (7.17) | 9.54 (7.15) | 8.12 (7.15) | 0.012 |
| Child's gender, n (%) | | | | 0.213 |
| Male | 361 (48.4) | 102 (44.7) | 259 (50.0) |  |
| Female | 385 (51.6) | 126 (55.3) | 259 (50.0) |  |
| Relationship to the child, n (%) | | | | 0.011 |
| Mother | 623 (83.5) | 178 (78.1) | 445 (85.9) |  |
| Father or others | 123 (16.5) | 50 (21.9) | 73 (14.1) |  |
| Child's Hukou, n (%) | | | | <0.001 |
| Shanghai | 412 (55.2) | 152 (66.7) | 260 (50.2) |  |
| Other provinces | 334 (44.8) | 76 (33.3) | 258 (49.8) |  |
| One-child or not, n (%) | | | | 0.409 |
| Yes | 516 (69.2) | 163 (71.5) | 353 (68.1) |  |
| No | 230 (30.8) | 65 (28.5) | 165 (31.9) |  |
| Caregiver's education, n (%) | | | | 0.007 |
| Below university | 151 (20.2) | 32 (14.0) | 119 (23.0) |  |
| University or higher | 595 (79.8) | 196 (86.0) | 399 (77.0) |  |
| Family monthly income per capita (in RMB), n (%) | | | | 0.183 |
| <4500 | 73 (10.9) | 14 (6.9) | 59 (12.7) |  |
| 4500~7500 | 161 (24.1) | 50 (24.8) | 111 (23.8) |  |
| 7500~12500 | 191 (28.6) | 62 (30.7) | 129 (27.7) |  |
| ≥12500 | 243 (36.4) | 76 (37.6) | 167 (35.8) |  |
| Source of parental information, n (%) | | | | 0.986 |
| Others | 64 (8.6) | 19 (8.3) | 45 (8.7) |  |
| Social media | 682 (91.4) | 209 (91.7) | 473 (91.3) |  |

Note. Values represent n (%) unless stated otherwise. ^a^*P* values for comparing the baseline characteristics between caregivers with different adherent status in the intervention group using *t*-test or chi-square test.

Table S2. Baseline characteristics of the participants with different retention status in the WOA-based intervention (April 7, 2020, to April 20, 2021 in Shanghai)

| Variable | Baseline | Lost to follow-up | Follow-up | *P*^a^ | *P*^b^ |
| --- | --- | --- | --- | --- | --- |
| No. of caregivers | 1332 | 249 | 1083 |  |  |
| Child's age (months), M (SD) | 8.74 (7.00) | 8.16 (6.73) | 8.87 (7.05) | 0.228 | 0.147 |
| Child's gender, n (%) | | | | 0.947 | 0.992 |
| Male | 645 (48.4) | 120 (48.2) | 525 (48.5) |  |  |
| Female | 687 (51.6) | 129 (51.8) | 558 (51.5) |  |  |
| Relationship to the child, n (%) | | | | <0.001 | <0.001 |
| Mother | 1088 (81.7) | 174 (69.9) | 914 (84.4) |  |  |
| Father or others | 244 (18.3) | 75 (30.1) | 169 (15.6) |  |  |
| Child's Hukou, n (%) | | | | 0.099 | 0.044 |
| Shanghai | 826 (62.0) | 140 (56.2) | 686 (63.3) |  |  |
| Other provinces | 506 (38.0) | 109 (43.8) | 397 (36.7) |  |  |
| One-child or not, n (%) | | | | 0.878 | 0.842 |
| Yes | 948 (71.2) | 179 (71.9) | 769 (71.0) |  |  |
| No | 384 (28.8) | 70 (28.1) | 314 (29.0) |  |  |
| Caregiver's education, n (%) | | | | 0.300 | 0.199 |
| Below university | 228 (17.1) | 50 (20.1) | 178 (16.4) |  |  |
| University or higher | 1104 (82.9) | 199 (79.9) | 905 (83.6) |  |  |
| Family monthly income per capita (in RMB), n (%) | | | | 0.670 | 0.530 |
| <4500 | 122 (10.2) | 22 (10.1) | 100 (10.2) |  |  |
| 4500~7500 | 287 (23.9) | 44 (20.2) | 243 (24.7) |  |  |
| 7500~12500 | 377 (31.4) | 71 (32.6) | 306 (31.2) |  |  |
| ≥12500 | 414 (34.5) | 81 (37.2) | 333 (33.9) |  |  |
| Source of parental information, n (%) | | | | 0.171 | 0.085 |
| Others | 97 (7.3) | 25 (10.0) | 72 (6.6) |  |  |
| Social media | 1235 (92.7) | 224 (90.0) | 1011 (93.4) |  |  |

Note. Values represent n (%) unless stated otherwise. ^a^*P* values for comparing baseline characteristics between caregivers enrolled at baseline and caregivers lost to follow-up using *t*-test or chi-square test. ^b^*P* values for comparing baseline characteristics between caregivers with different retention status using *t*-test or chi-square test.
