# Supplementary material for: Improving Parental Health Literacy in Primary Caregivers of 0- to 3-Year-Old Children Through a WeChat Official Account: Cluster Randomized Controlled Trial
Source: JMIR Public Health Surveill. 2024 Jul 4;10:e54623. doi: 10.2196/54623 (PMC11238142; doi:10.2196/54623)
Supplement: Multimedia Appendix 2 [file publichealth-v10-e54623-s002.docx]

## Multimedia appendix 2. Missing data report for the WOA-based intervention

Table 1 Missing data report for the WOA-based intervention (April 7, 2020, to April 20, 2021 in Shanghai)

| Outcome | Eligible | Available | | Missing  Rate (%) |
| --- | --- | --- | --- | --- |
|  |  | Baseline | Final |  |
| **CPHLQ^a^** | 1332 | 1332 | 1083 | 18.7 |
| **Anthropometry^b^** | 1332 | 1332 | 1253 | 5.9 |
| **Parenting behaviors** |  |  |  |  |
| EBF at 6 months^b^ | 312 | NA | 290 | 7.1 |
| BF at 12 months^b^ | 411 | NA | 363 | 11.7 |
| Supplementation of Iron-fortified staple foods at 6 months^b^ | 525 | NA | 458 | 12.8 |
| Routine checkup of child's oral health^a^ | 1332 | NA | 1081 | 18.8 |
| Routine checkup of child's vision health^a^ | 1332 | NA | 1081 | 18.8 |
| **Awareness of VD supplementation for infants aged 0 to 6 months^a^** | 1332 | NA | 1054 | 20.9 |
| **Children's health outcomes** |  |  |  |  |
| Unintended injuries^a^ | 1332 | NA | 1081 | 18.8 |
| Hospitalization^a^ | 1332 | NA | 1081 | 18.8 |
| Anemia^b^ | 1332 | 1332 | 1314 | 1.4 |
| Obesity or overweight^b^ | 1332 | 1332 | 1308 | 1.8 |
| Diarrhea^a^ | 1332 | NA | 1081 | 18.8 |

Note. Values represent number of participants unless stated otherwise. ^a^Data collected from online surveys through WOA. ^b^Data collected from routine health check-up records. CPHLQ=Chinese Parental Health Literacy Questionnaire; EBF=exclusive breastfeeding; BF=breastfeeding; VD=vitamin D; NA=not applicable.
